# Topics of Public Interest

**Published:** 1914-02

**Authors:** 


### TOPICS OF PUBLIC INTEREST.

STANDARDIZED DEATH RATE FOR STATES AND CITIES.

| AREA. | 1912 | 1911 |
|---|---|---|
| Registration states | 13.5 | 13.7 |
| California | 13.2 | 12.8 |
| Colorado | 12.2 | 13.6 |
| Connecticut | 14.3 | 14.8 |
| Indiana | 12.4 | 12.3 |
| Kentucky* | 13.2 | 13.4 |
| Maine | 12.6 | 13.0 |
| Maryland* | 15.7 | 15.9 |
| Massachusetts | 14.7 | 15.0 |
| Michigan | 12.6 | 12.4 |
| Minnesota | 9.8 | 10.8 |
| Missouri | 12.6 | 13.1 |
| Montana | 11.5 | 11.6 |
| New Hampshire | 13.6 | 14.2 |
| New Jersey | 14.5 | 15.1 |
| New York | 15.2 | 15.7 |
| Ohio | 15.7 | 12.4 |
| Pennsylvania | 14.1 | 14.4 |
| Rhode Island | 15.4 | 15.7 |
| Utah | 10.6 | 11.0 |
| Vermont | 12.2 | 12.6 |
| Washington | 8.7 | 9.8 |
| Wisconsin | 10.8 | 11.0 |

CITIES OF 100,000 POPULATION OR OVER IN 1910.

| | | |
|---|---|---|
| Birmingham, Ala* | 20.4 | 21.3 |
| Los Angeles, Cal | 14.6 | 14.3 |
| Oakland, Cal | 12.5 | 12.5 |
| San Francisco, Cal | 16.4 | 15.9 |
| Denver, Colo | 15.1 | 16.5 |
| Bridgeport, Conn | 15.4 | 15.4 |
| New Haven, Conn | 16.8 | 17.1 |
| Washington, D. C.* | 18.5 | 18.9 |
| Atlanta, Ga.* | 20.0 | 22.0 |
| Chicago, Ill. | 16.7 | 16.4 |
| Indianapolis, Ind. | 15.6 | 15.3 |
| Louisville, Ky.* | 17.4 | 17.0 |
| New Orleans, La.* | 21.5 | 21.8 |

| | | |
|---|---|---|
| Baltimore, Md.* | 19.1 | 19.4 |
| Boston, Mass. | 17.2 | 17.9 |
| Cambridge, Mass. | 13.1 | 15.4 |
| Fall River Mass. | 17.2 | 18.4 |
| Lowell, Mass. | 18.4 | 18.8 |
| Worcester, Mass. | 16.7 | 16.2 |
| Detroit, Mich. | 17.2 | 16.0 |
| Grand Rapids, Mich. | 13.1 | 13.7 |
| Minneapolis, Minn. | 11.6 | 12.9 |
| St. Paul, Minn. | 12.0 | 12.9 |
| Kansas City, Mo. | 16.7 | 16.9 |
| St. Louis, Mo. | 16.2 | 16.8 |
| Omaha, Neb. | 14.5 | 15.8 |
| Jersey City, N. J. | 15.5 | 17.6 |
| Newark, N. J. | 15.6 | 16.2 |
| Paterson, N. J. | 15.3 | 15.9 |
| Albany, N. Y. | 19.5 | 19.7 |
| Buffalo, N. York | 16.1 | 15.8 |
| New York, N. Y. | 16.4 | 17.2 |
| Rochester, N. Y. | 15.3 | 15.1 |
| Syracuse, N. Y. | 15.8 | 14.9 |
| Cincinnati, Ohio | 16.9 | 16.9 |
| Cleveland, Ohio | 15.2 | 15.3 |
| Columbus, Ohio | 15.3 | 15.2 |
| Dayton, Ohio | 15.4 | 14.0 |
| Toledo, Ohio | 16.6 | 15.6 |
| Portland, Ore. | 11.0 | 12.6 |
| Philadelphia, Pa. | 16.1 | 17.4 |
| Pittsburgh, Pa. | 18.0 | 16.9 |
| Scranton, Pa. | 16.5 | 16.7 |
| Providence, R. I. | 16.4 | 16.2 |
| Memphis, Tenn.* | 23.9 | 23.4 |
| Nashville, Tenn.* | 20.8 | 22.1 |
| Richmond, Va.* | 22.8 | 23.2 |
| Seattle, Wash. | 9.6 | 10.4 |
| Spokane, Wash. | 9.9 | 13.7 |
| Milwaukee, Wis. | 14.6 | 13.3 |

* Cities with large proportion of colored population.

---

TWENTY-NINE COUNTIES PROVIDED (OR ABOUT TO BE) WITH TUBERCULOSIS HOSPITAL. The following table shows the counties of New York State, arranged according to ther population, and gives the assessed valuation of each and the average annual number of deaths from tuberculosis.

The 29 counties printed in heavy type either have public tuberculosis hospitals within their borders or have voted, through their boards of supervisors, to establish county tuberculosis hospitals.

| Rank in population | County | Population 1910 | Assessed valuation | Average annual No. tuberculosis deaths in last 7 years |
|---|---|---|---|---|
| 1 | ERIE | 528,985 | $386,204,382.00 | 602 |
| 2 | MONROE | 283,212 | 237,223,974.00 | 317 |
| 3 | WESTCHESTER | 283,055 | 358,215,745.00 | 357 |
| 4 | ONONDAGA | 200,298 | 181,251,726.00 | 205 |
| 5 | ALBANY | 173,066 | 134,660,347.00 | 316 |
| 6 | ONEIDA | 154,157 | 77,340,478.00 | 195 |
| 7 | RENSSELAER | 122,276 | 84,974,673.00 | 234 |
| 8 | ORANGE | 116,001 | 51,898,881.00 | 169 |
| 9 | Chautauqua | 105,126 | 56,200,931.00 | 71 |
| 10 | SUFFOLK | 96,138 | 87,051,236.00 | 88 |
| 11 | NIAGARA | 92,036 | 68,981,495.00 | 82 |
| 12 | ULSTER | 91,769 | 29,486,275.00 | 130 |
| 13 | St. Lawrence | 89,005 | 45,775,940.00 | 94 |
| 14 | SCHENECTADY | 88,235 | 62,840,713.00 | 89 |
| 15 | DUTCHESS | 87,661 | 59,645,968.00 | 107 |
| 16 | Nassau | 83,930 | 97,203,283.00 | 78 |
| 17 | STEUBEN | 83,363 | 45,136,921.00 | 58 |
| 18 | JEFFERSON | 80,382 | 47,424,484.00 | 65 |
| 19 | BROOME | 78,000 | 47,173,355.00 | 76 |
| 20 | OSWEGO | 71,664 | 32,575,051.00 | 65 |
| 21 | CAYUGA | 67,106 | 41,439,936.00 | 68 |
| 22 | CATTARAUGUS | 65,919 | 28,178,830.00 | 43 |
| 23 | SARATOGA | 61,917 | 27,584,152.00 | 75 |
| 24 | MONTGOMERY | 57,567 | 29,205,221.00 | 63 |
| 25 | HERKIMER | 56,356 | 31,103,266.00 | 54 |
| 26 | CHEMUNG | 54,662 | 31,305,936.00 | 56 |
| 27 | ONTARIO | 52,286 | 35,365,978.00 | 41 |
| 28 | Wayne | 50,179 | 27,586,328.00 | 38 |
| 29 | CLINTON | 48,230 | 9,794,319.00 | 53 |
| 30 | Washington | 47,778 | 20,920,253.00 | 48 |
| 31 | Otsego | 47,216 | 24,613,186.00 | 32 |
| 32 | Rockland | 46,873 | 31,619,675.00 | 50 |
| 33 | Franklin | 45,717 | 12,738,959.00 | 159 |
| 34 | Delaware | 45,575 | 15,890,010.00 | 32 |
| 35 | FULTON | 44,534 | 16,518,523.00 | 46 |
| 36 | Columbia | 43,658 | 27,345,896.00 | 54 |
| 37 | Allegany | 41,412 | 19,127,101.00 | 19 |
| 38 | Madison | 39,289 | 21,325,848.00 | 29 |
| 39 | Livingston | 38,037 | 28,375,199.00 | 33 |
| 40 | Genesee | 37,615 | 26,751,482.00 | 27 |
| 41 | CHENANGO | 35,575 | 16,720,005.00 | 27 |
| 42 | Sullivan | 33,808 | 7,142,055 00 | 159 |
| 43 | TOMPKINS | 33,647 | 20,612,808.00 | 28 |
| 44 | Essex | 33,458 | 12,744,085.00 | 46 |
| 45 | WARREN | 32,223 | 11,252,330.00 | 35 |

| | | | | |
|---|---|---|---|---|
| 46 | Orleans | 32,000 | 19,015,329.00 | 33 |
| 47 | Wyoming | 31,880 | 17,530,849.00 | 19 |
| 48 | Greene | 30,214 | 12,947,633.00 | 50 |
| 49 | Cortland | 29,249 | 17,086,013.00 | 19 |
| 50 | Seneca | 26,972 | 16,613,416.00 | 25 |
| 51 | Tioga | 25,624 | 14,248,180.00 | 19 |
| 52 | Lewis | 24,849 | 10,812,384.00 | 17 |
| 53 | Schoharie | 23,855 | 11,477,535.00 | 22 |
| 54 | Yates | 18,642 | 11,526,407.00 | 14 |
| 55 | Putnam | 14,665 | 14,173,376.00 | 17 |
| 56 | Schuyler | 14,004 | 6,797,222.00 | 10 |
| 57 | Hamilton | 4,373 | 4,879,042.00 | 4 |

—S. C. C. A. News, December, 1913.

CURE OF GONORRHOEA BY HEAT. J. A. Fulton of Astoria, Wash., *Northwest Med.*, December, 1913, refers to his initial report in the *Med. Rec.*, February 12, 1912 and to the report by Majors L. W. Harrison and C. J. Stoughton, in the Jour. Royal Army Med. Corps, February, 1913 which we abstracted some months ago. Naturally, Fulton claims priority which we are glad to accord, subject to possible conflicting claims. He uses a temperature of 110–120 F., for 20-30 minutes, by means of a catheter with inlet and outlet tubes and a thermometer in each. One or two treatments of acute cases usually produce negative bacteriologic findings.

The following bill is proposed to be introduced into the New York Legislature of 1914, by the Society for the Prevention of Abuse in Animal Experimentation, 204 Montague Street, Brooklyn, N. Y.

An act to create a commission to investigate and report upon the condition of the practice of human and animal experimentation in the State of New York, to show what regulations are necessary to prevent cruelty to human beings or animals; and likewise to prevent any abuse of or interference with the private rights of human beings in our charitable institutions and elsewhere, by experimentation upon them without authority and consent.

The people of the State of New York represented in Senate and Assembly, do enact as follows:

SECTION I. The Governor is hereby empowered and directed to appoint a commission which shall consist of five (5) members, two (2) of whom shall be physicians or persons experienced in the practice of vivisection and residing within this state, two (2) of whom shall be active members of some organization within this state having for its purposes the prevention of cruelty but who shall not be physicians, and the remaining one (1) member of which commission shall be a lawyer residing within this state.

SEC. II. Such commission shall fully investigate and report upon:

(a) The present condition and extent of the practice of experimentation upon human beings without their consent; especially upon children and other patients in hospitals, public institutions or elsewhere within this state by inoculation or by any other form of treatment or tests not undertaken for the direct benefit of the individuals experimented upon and not having relation to their individual necessities. It shall also report what further laws are necessary to protect such persons from any injury or any interference with their personal rights by such practice or by the abuse thereof.

(b) It shall investigate and report upon the condition and extent of the practice of experimentation on living animals in the state and upon the amount of avoidable cruelty or suffering involved therein; and shall also make a full inquiry into the condition of the law of this state for the protection, regulation and license of scientific investigation or research of this character by competent experts. It shall also consider the condition and effectiveness of the law for the prevention of abuse in such practice. It shall inquire what further legislation, may be needed to prevent unnecessary suffering of animals through such practice or through its abuse and, also, properly to license and limit legitimate scientific experimentation to experts of approved competency.

Sec. III. For the purpose of this investigation the said commission is hereby authorized and empowered to subpœna witnesses; to send for persons or papers to administer oaths and to examine witnesses and papers respecting all matters pertaining to this subject. It shall be authorized to employ necessary clerical or other assistants. For this purpose the sum of five thousand dollars or so much thereof as is necessary is hereby appropriated.

This commission shall serve without compensation, and shall make a full and final report to the Governor, including such recommendations for legislation as in its judgment seem proper within one year after its appointment.

Sec. IV. This act shall take effect immediately.

---

The American College of Surgeons has been submitted to so much criticism that we take pleasure in reviewing the circular sent by the Secretary, Dr. Franklin H. Martin of Chicago, from which we are authorized to extract. The impression that the college might interfere with vested rights to practice medicine in its entirety seem to have been based on the call to the organization meeting in Washington, in May 1913:

"First. It should formulate a minimum standard of requirements which should be possessed by any authorized graduate in medicine who is allowed to perform, independently, surgical operations in general surgery or any of its specialties.

"Second. It should consider the desirability of listing the names of those who desire to practice surgery and who come under the authorized requirements.

"Third. It should seek the means of legalizing under national, colonial, state, or provincial laws, a distinct degree supplementing the medical degree which shall be conferred upon physicians possessing the requirements recognized by this law as necessary to be possessed by operating surgeons.

"Fourth. It should seek co-operation with the medical schools of the continent which have the right to confer the degree of M. D. under the present recognized standards, and urge these colleges to confer a supplementary degree on those of its graduates who have, in addition to their medical course, fulfilled the necessary apprenticeship in surgical hospitals, operative laboratories, and actual operative surgery.

"Fifth. It should authorize and popularize the use of this title by men upon whom it is conferred, and its use should especially be urged in all directories of physicians in order that the laity as well as medical men may distinguish between the men who have been authorized to practice surgery and those who have not."

As formally recorded in the by-laws, it will be observed that criticism on this score is not justified unless it shall develop that the College, while maintaining the high standard appropriate to a select society endeavors to control the license to practice surgery or to exploit the privilege of membership to an undue degree.

II. Object. The object of the College shall be to elevate the standard of surgery, to establish a standard of competency and of character for practitioners of surgery, to provide a method of granting fellowships in the organization, and to educate the public and the profession to understand that the practice of surgery calls for special training, and that the surgeon elected to fellowship in this College has had such training and is properly qualified to practice surgery.

We find nothing either in the circular of information nor in the way of designations after the published list of members to justify the impression—which, however, was warranted by the preliminary announcements—that members are to be arranged in classes of distinction. Over 2000 applications mostly voluntary, were received up to November, 1913, of which number 1057 were recommended for membership and many more probably will be favorably passed upon. Convocations for the edmission of fellows will be held in some eastern city on Monday evening of the week of the A. M. A. meeting (June) and at some place to be designated later, in November. At a rough estimate, there are about 5000 surgeons, in the usual special sense, in the United States. While the College follows a broad policy in admitting members from various specialties which are more or less surgical, it seems reasonable to suppose that the great majority of its fellows will be surgeons in the ordinary sense of the word and that other specialists will not seek membership in any sense as a pre-requisite to practice and, so far as can be judged from scanning the list of fellows already elected, the great majority are strictly surgeons. It is obvious that, at the inception of so large a movement, special pains must be taken in the selection and, on the other hand, that a great many men would, on account of conservatism, delay their applications. The mere fact that approximately a fifth of the total surgical strength of the country has already been enrolled, indicates both a strong professional sentiment in favor of such an organization and a disposition to deal fairly with the surgeons of the country.

We believe that neither the medical press nor individuals should anticipate the actual development of this organization. Before criticising, let us wait and see whether its policy is to require special eminence for admission or whether all bona fide surgeons, in good professional standing, are to be admitted. Either policy is perfectly proper and supported by precedent, but each, obviously, must influence the future policy of the organization. After the College has definitely embarked on one of these two courses, further criticism should be deferred until it has been shown by experience that the former policy is being used to the disadvantage of the general body of surgeons or that the latter is being used to interfere with the legal rights of practitioners as a whole.

In accepting and publishing the address of its President, Dr. J. M. T. Finney, the College has put itself plainly on record as opposed to graft, favoritism or mercenary motives, the establishment of a surgical trust or the control of politics. While the question of the necessity of such an institution allows a difference of opinion and while there may be honest differences of opinion as to details, in its subsequent management, the hostile criticisms that have been expressed imply a diffusion of insincerity that is not only inconceivably base but suicidal. We ask, therefore, not only a fair, judicial attitude but a cordial welcome to this new institution.

---

Scarlet Fever Germ. On investigation of the report that Dr. Terry of Detroit had identified a specific germ for this disease, we are informed that the press notices are premature and that while Dr. Terry has made some valuable observations, he is not making any such claim at present.

---

Wasserman Test. Beginning with 1914, the Health Department of Buffalo will make this test free of charge for physicians, on any local case. Special outfits and a detailed circular of information have been prepared. Samples of blood should be sent in Monday of each week, Tuesday being devoted to making the tests.

---

Grain Trade of Buffalo. Mr. Junius S. Smith, Lake Weighmaster of the Corn Exchange of Buffalo (the word corn being used in the original English sense) reports that over 192 million bushels of grain were received at Buffalo in 1913, carried by 998 lake boats. Over a third came from Canada. Nearly 17 million bushels were held in cargo, January 1. The grain carriage by lake has been reduced to such a system that the discrepancy between the weight as delivered to and extracted from the boats is only about one part in 10,000, about one tenth what it was when Mr. Smith undertook the work in 1872.

---

Hospital Rates in Lockport. An attempt has been made to reduce the charges for ward service to non-residents from $12 to $8 a week, on account of the prevalence of the lower rate in Buffalo. The Common Council, late in December, refused to reduce the rate.

---

The Broad St. Hospital of Oneida, opened March 1, 1907 with 25 beds and enlarged in 1911 to about 30 beds, will be further enlarged to a capacity of 60 patients, work to be begun about March 1.

---

Opening for Physician. The Hospital Board of St. Louis is looking for a medical man of wide experience, as medical officer at the workhouse. He is expected to devote all his time to the work, sleep with the prisoners and eat with the guards. The salary is $100 per month. An examination to have been held November 29, has been postponed as no applications were received. Comment is withheld, on account of post office regulations.

---

The Court of Appeals of Louisville has awarded the father of a child $250 damages against the Cumberland Telephone and Telegraph Company for failing to reach a physician, the child dying of diphtheria. Probably a case of "Excuse it, please, I

will call you later." By the way, did anyone ever receive that call later?

---

The Automobile License Law has been declared unconstitutional in Ohio on the ground of class legislation and licenses and number plates are being withheld pending decision of the Supreme Court. We believe few will object to the payment of the tax, anywhere, if the proceeds are used legitimately for road improvement. Except brick roads, most state roads with which we are familiar are tire destroyers.

---

The Same Problem in Ethics. In our January issue, we alluded to a scathing criticism of noted surgeons for what seemed to us merely the enterprise of a passenger agent of a railroad but the matter served as a text for discussing whether the publicity of well known men might not influence the ethical conduct of less fortunate practitioners. Last year we noted the action of a Baltimore society condemning news notices of operations or other hospital treatment, in which the names of attendants were published and we called attention to the injustice of enforcing the theoretically correct principle involved, to its literal extreme. Now, formal inquiry has been addressed to a celebrated surgeon on account of publicity in connection with radium. So far as radium is concerned, we can see no reason for the somewhat spectacular items which have appeared in newspapers. Except for the gradual accumulation of experience, in both a positive and a negative direction, and certain interesting studies along the lines of radio-activity generally and the possibility of transmutation of what have been regarded as elements, there has been no conspicuous advance in the last six years. No epoch-making discovery has been made to justify newspaper publicity and, if anything, the general experience should lead rather to a hopeful conservatism and guarded use of radio-activity than to a disuse of accepted surgical measures against malignant diseases. Even if the contrary were true, the ethical status of the problem would remain the same. To facilitate discussion, the following somewhat dogmatic and conflicting statements may be made: 1. The ethical conduct of the great man should not differ essentially from that of the mediocre man, in any profession. 2. Progress should not be obstructed by ethics. 3. Proper, spontaneous interest in a man, due to genuine merit or unavoidable publicity, should not be considered to detract from his ethical standing. We do not know of any way to harmonize the apparent inconsistency except by a general recourse to sincerity and unselfishness.

COCAINE. In the October issue, we published a full report on the New York State Law, feeling confident that this one article would be worth in dollars and cents, all that our subscribers pay for the Journal in a year. Failure to heed our warning has, up to date resulted as follows: one year in the penitentiary for one physician, one other and a druggist held for grand jury—for Buffalo alone. We do not, however, claim this result as proof of the value of the article as the evidence showed that the loss of time and expense were not due to any technical violation of the law. The law is drastic, based on stern necessity to control a great evil. Its requirements are troublesome and not entirely such as would occur to conscientious men. We, therefore urge careful reference to the article to avoid technical infractions.

---

AMBULANCE SERVICE. The Buffalo General Hospital reports, for the last fiscal year, 1526 trips, averaging 34 minutes each. A general average of over 4 trips a day was maintained, the highest number for any one day being 12, representing 6 hours work.

THE HYDROPHOBIA GERM has been discovered by Noguchi and classed as a protozoon. Barring contradictory findings by others, this should put an end to the dispute as to whether hydrophobia is a specific disease, on which question, we have always preserved a judicial attitude. We believe, however, that it must be conceded that purely clinical diagnosis of rabies must have included cases of tetanus and of hysteria major.

---

RAPE. The five-year-old daughter of a North Carolina physician was recently assaulted by a negro who was spirited away to jail, to prevent a lynching.

---

EFFICIENCY OF ANTI-TYPHOID VACCINATION. But two cases of typhoid occurred in the army in 1913, as compared with an average of about 250 before vaccination was practiced. In the navy, in which compulsory vaccination was not at first practiced, seven cases occurred in a total strength of 50,000, four of these being at a remote tropical station where the vaccine had probably deteriorated. While sanitation and education must be given credit, it is inconceivable that such striking results could have been obtained without a genuine protective power on the part of the vaccination.

---

THE VALUE OF EDUCATION—A NEGATIVE PROOF. At few places has the uselessness of vaccination against variola and the right of the individual to resist compulsory laws, been so thoroughly and influentially taught by a medical authority as at Niagara Falls. At few places has there been, relatively to the population, so much opportunity for importation of the germ and at few has the population so general an economic interest in freedom from small pox, or any other disease which would lead to quarantine or voluntary restriction of travel to a given point. Post hoc, if not proter hoc, Niagara Falls now has an epidemic of over 200 cases and is enjoying a quarantine and nearby towns are also suffering. So far as we can learn, not a single case has developed in a person theoretically immunized, according to accepted standards. We hope to publish a complete report of the epidemic when it is practically at an end and meantime, emphasize the opportunity for testing the conclusions of Fornet of Berlin abstracted at some length in our issue of December 1913. Anti-vaccinationists are supposed to be opposed to human experimentation. Here, we have human experimentation carried on on a comparatively large scale. We trust that the health authorities will make a full investigation, with accurate statistics as to the incidence of and escape from variola, in the vaccinated and the unvaccinated, with such opportunities for argument from the antis that this experiment may never need to be tried again.

---

Income Tax Correction. The statements in the editorial on this subject in the January issue, regarding estimate of income from homes owned by the occupant, should be corrected in accordance with recent rulings. Neither value as rental nor expenses except for taxes, are to be counted in calculating net incomes. This ruling is wholesome, as it prevents a good deal of confusion and it places a small premium on home-owning, which is a good thing for the community.

---

Radium Missing. 35 milligrams of radium, worth $4500, is reported missing from Chicago since the holidays. The warning is published in the daily papers that, unless the radium is properly protected, it may kill the thief.

---

Broome County Tuberculosis Hospital. The supervisors have voted to buy the Mountain Sanitarium property of the Binghamton City Hospital and to conduct it, beginning January 1, 1914, as a county hospital for tuberculosis. The site contains 30 acres and is valued at $10,000.

---

Dr. George E. Smith of Fredonia has received a verdict of no cause of action in a suit brought against him for malpractice.

THE FLOWER HOSPITAL, 450 E. 64, N. Y., opened a new pavilion for private patients, January 17. The rates vary from $2 for ward beds to $25 for suite of two rooms with bath, by the day.

---

ERIE COUNTY PHYSICIANS. County Commissioner William Hunt has appointed Dr. Frederick M. Boyle, County Physician at a salary of $3000; Drs. George B. Stocker and Bruce L. B. Cook, Deputy Physicians at salaries of $2500.

---

DENTAL DISPENSARIES FOR BUFFALO. Health Commissioner Fronczak's efforts in this direction for the last couple of years have been crowned with success.

This is a list of new positions and increases in the present force approved yesterday by the committee:

Ten school nurses at $720 a year.

Two dental inspectors at $500 each.

Two dentists at $1,000 each.

Two assistant dentists at $400 each.

One examiner in psycho-physiological division, $1,000.

Two diagnosticians at $2,000 each.

Two clerks at $900 each.

Three inspectors of food and drugs at $1,000 each.

One clerk and stenographer at $1,000.

One permit and filing clerk at $900.

One Italian speaking tenement and lodging-house inspector at $1,000.

Considerable opposition was made to the project of establishing dental dispensaries and one of the daily papers heads its report "Creates Lot of New Jobs." With all consideration for the rights of tax payers and conservatism in regard to paternal methods, there is a real need for dispensaries along all medical lines but they should be carefully supervised to prevent their use by persons able to pay for treatment.

---

DEATHS IN PROFESSIONAL OFFICES. Recently, two such cases have occurred in western New York, both of women, one following a hypodermatic injection of peptonate of iron by a regular practitioner, the other during an abortion apparently caused by gossypium, in the office of a mechano-therapist. In the former case, blame was placed at the inquest on the physician for having used a method not thoroughly established. (Note: The Editor has a vial of tablets of citrate of iron prepared for this purpose some years ago but never used, as it was concluded that there was no special indication for administering iron in this way and some apprehension of results existed. However, one who has come so near the same method can sympathize with the unfortunate one who carried it out.) In the latter case, up to the date of writing, no evidence has appeared to incriminate the mechano-therapist in any way and he is held on the nominal charge of practicing medicine without a license which charge, we believe, can scarcely be upheld under the existing law.

---

GIRLS PREFERRED FOR ADOPTION. The State Charities Aid Association, 105 E. 22, N. Y., reports that of 4,754 applications for children from orphan asylums in 15 years, 3,011 specified girls, 190 were indifferent and 30 would take both a girl and a boy. This can scarcely be considered to represent a prejudice against male offspring, as persons willing to adopt orphans are usually lacking in keen family pride and wish children mainly for domestic association, in which girls would naturally be preferred. The report gives the following interesting statistics:

| | |
|---|---|
| Legally adopted | 628 |
| Returned to relatives of good character who had become able to care for them | 44 |
| Returned to agencies or institutions from which the children were received | 43 |
| Died | 49 |
| Became of age and self-supporting | 51 |
| Transferred to and placed through other societies and institutions | 10 |
| Placed in institutions for special treatment | 25 |
| Married | 6 |
| Remaining under the oversight of the S. C. A. A. | 928 |
| Total | 1,784 |

---

CHARGES have been brought against the Superintendent of the Gowanda State Hospital, of drunkenness and neglect of duty. The provisional defense is that the charges are due to desire for vengeance by an employe discharged for cause and the latter seems to have been desirous of withdrawing his charges but they are being pushed by others.

---

FEES FOR REPORTING COMMUNICABLE DISEASES. The State Health Commissioner has ruled that Dunkirk physicians are still entitled to receive a fee of 25 cents, in spite of recent changes in the law. We understand that this ruling is applicable to other places.

---

The Ontario County Laboratory has recently been much enlarged. The addition includes a consulting room, pathology room, autopsy or experimental room, animal room and the necessary storage and toilet space. This is the first county laboratory to be established in the United States and it is gratifying to know that the work has demanded an addition. The equipment is adequate for all modern diagnostic work and for research on a small scale.

---

Typhoid Epidemic. Centralia, Wash., population 10,000, developed 300 cases in three weeks, last month, 16 dead at time of report. The water supply was from driven wells but with an emergency supply from a creek, and hence infection from the water shed. A very interesting case of human experimentation well illustrating the antivivisectionist argument that a cruel experiment should not be repeated to support a fact already well established.

---

New York State Mortality 1913, 14.9:1000, against 14.8 for 1912, and, with this exception, the lowest on record.

